# Publisher Correction to: Changes in mental health during three waves of the COVID-19 pandemic: a repeated cross-sectional study among Polish university students

**DOI:** 10.1186/s12888-021-03680-7

**Published:** 2022-01-19

**Authors:** Aleksandra M. Rogowska, Dominika Ochnik, Cezary Kuśnierz, Karolina Chilicka, Monika Jakubiak, Maria Paradowska, Luiza Głazowska, Dawid Bojarski, Julia Fijołek, Marcin Podolak, Maciej Tomasiewicz, Dominika Nowicka, Marek Kawka, Maksymilian Grabarczyk, Zuzanna Babińska

**Affiliations:** 1 Institute of Psychology, University of Opole, Opole, Poland; 2 Faculty of Medicine, University of Technology, Katowice, Poland; 3 Faculty of Physical Education and Physiotherapy, Opole University of Technology, Opole, Poland; 4 Institute of Health Sciences, University of Opole, Opole, Poland; 5 Faculty of Economics, Maria Curie-Sklodowska University in Lublin, Lublin, Poland; 6 Faculty of Psychology and Cognitive studies, Adam Mickiewicz University in Poznań, Poznań, Poland; 7 Institute of Psychology, University of Lodz, Łódź, Poland; 8 Faculty of Medicine, Poznan University of Medical Sciences, Poznań, Poland; 9 Faculty of Medicine, Wroclaw Medical University, Wrocław, Poland; 10 Faculty of History, University of Warsaw, Warszawa, Poland; 11 Faculty of“Artes Liberales”, University of Warsaw, Warszawa, Poland; 12 Institute of the Middle and Far East, Faculty of International and Political Studies, Jagiellonian University, Kraków, Poland

**Correction to: BMC Psychiatry 21, 627 2021**

**https://doi.org/10.1186/s12888-021-03615-2**

Following the publication of the original article [1], the authors identified that the captions were incorrectly assigned to Figs. 3, 4, and 5. The figures with the correct captions are given below.

**Fig. 3:**
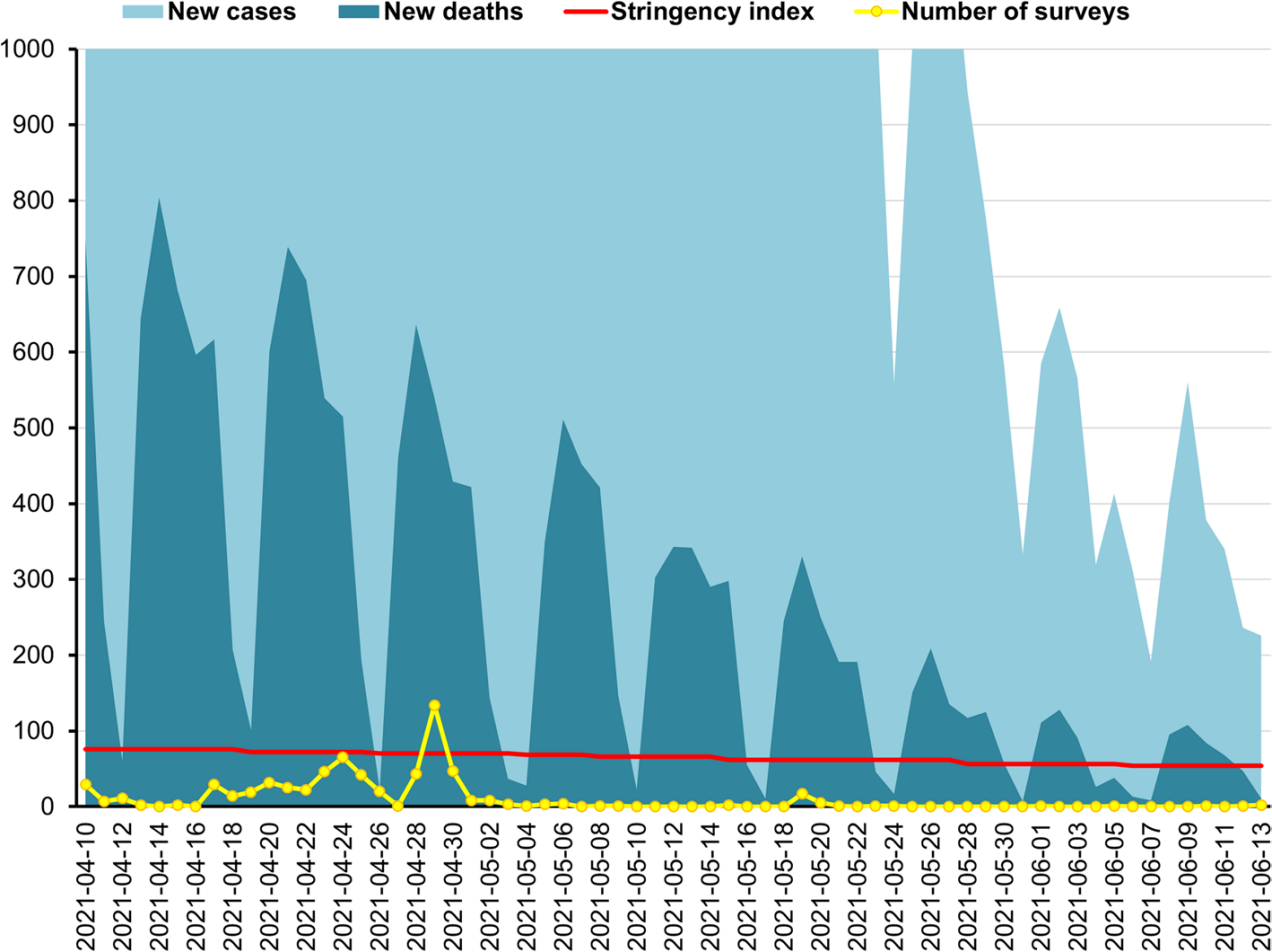
The third wave of the COVID-19 pandemic in Poland, between 10th April and 12 June 2021: New cases of Coronavirus ranged 192–24,892 daily (*M* = 5376.35, *SD* = 6179.14); New deaths from Coronavirus ranged 7–804 daily (*M* = 263.78, *SD* = 233.22); Stringency index ranged 53.7–75.93 (*M* = 65.09, *SD* = 7.41); Number of surveys ranged 0–134 daily (*M* = 10.03, *SD* = 21.30). Source of new cases, new deaths, and stringency index during the COVID-19 pandemic in Poland: Johns Hopkins University Center for Systems Science and Engineering (CSSE) COVID-19 Data [40]

**Fig. 4:**
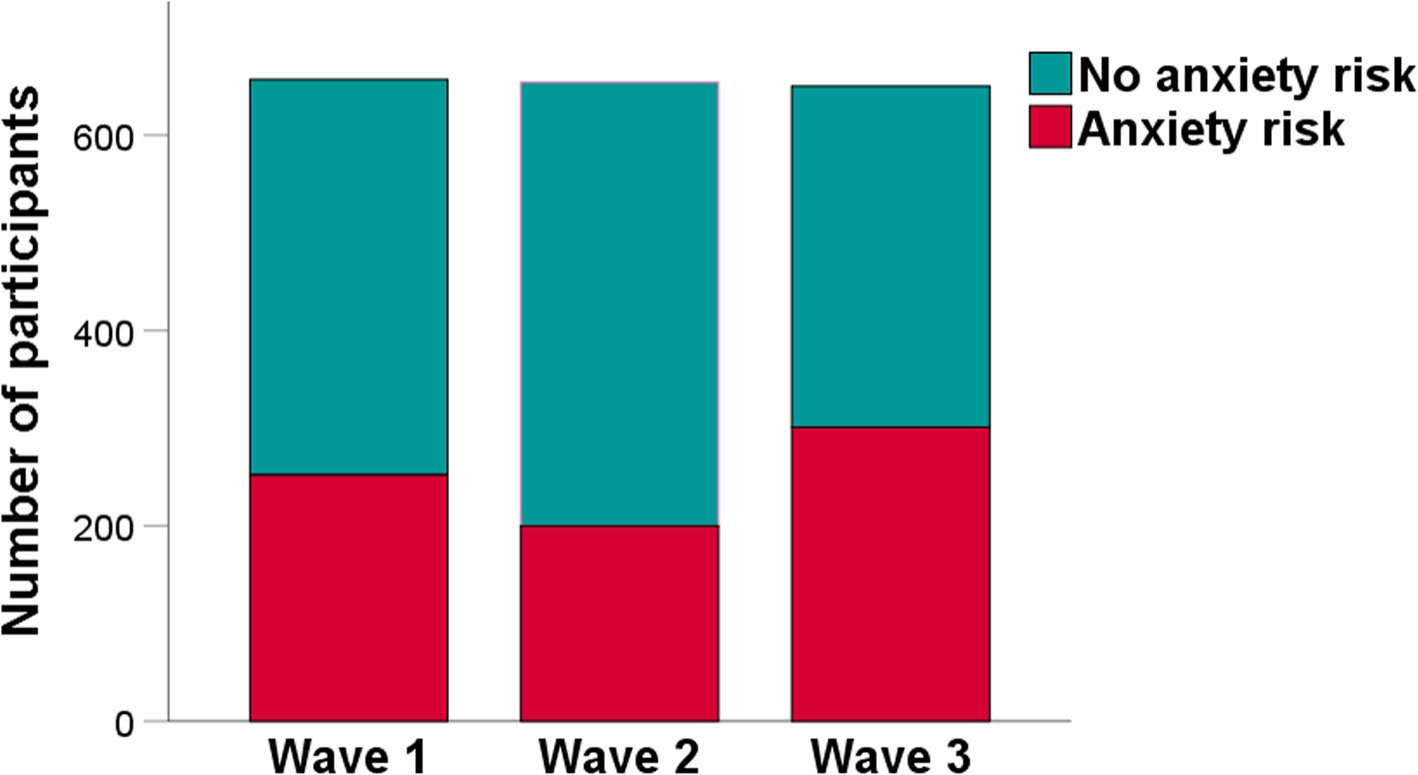
Frequency of people with anxiety risk during three waves of the COVID-19 pandemic

**Fig. 5:**
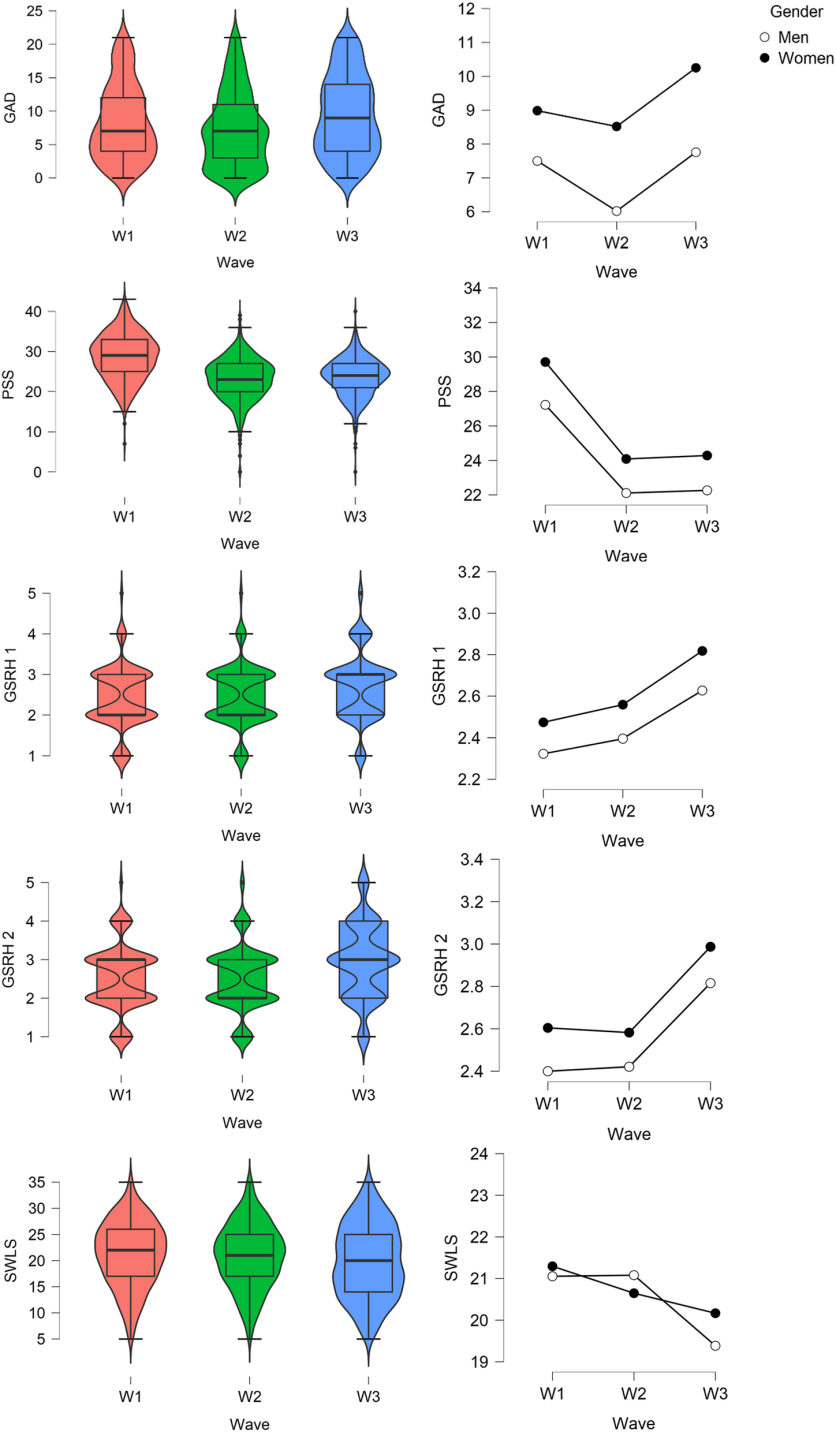
Boxplots (on the left, for the total sample) and linear plots (on the right, for gender differences), presenting scores of university students in anxiety (GAD), perceived stress (PSS), physical health (GSRH 1 and GSRH 2), and life satisfaction (SWLS) during the three waves of the COVID-19 pandemic

The publisher apologizes to the authors and readers for the inconvenience.

The original article [1] has been corrected.
